# Beliefs, attitude, and knowledge of the Iranian physiatrists towards neuromusculoskeletal ultrasound and common barriers in its application

**DOI:** 10.1186/s12891-020-03708-1

**Published:** 2020-10-14

**Authors:** Leyla Khodadadi, Afshin Karimzade, Seyed Mansoor Rayegani, Nafisseh Jafarian, Seyed Ahmad Raeissadat, Sina Yasrebi, Arash Babaei-Ghazani, Bina Eftekharsadat

**Affiliations:** 1 Physical Medicine and Rehabilitation Research Center and Department, Shaheed Beheshti University of Medical Sciences, Tehran, Iran; 2 Department of Physical Medicine and Rehabilitation, Shaheed Beheshti University of Medical Sciences, Tehran, Iran; 3 Physical Medicine and Rehabilitation Research Center, Shohada-e-Tajrish Hospital, Shaheed Beheshti University of Medical Sciences, Tehran, Iran; 4 Neshat Physical Medicine, and Rehabilitation Clinic, Karaj, Iran; 5 Department of Physical Medicine and Rehabilitation, Neuromusculoskeletal Research Center, School of Medicine, Iran University of Medical Sciences, Tehran, Iran; 6 Physical Medicine and Rehabilitation Research Center, Tabriz University of Medical Sciences, Tabriz, Iran

**Keywords:** Neuromusculoskeletal sonography, Physical medicine and rehabilitation, Knowledge assessment, Residency curriculum perspectives

## Abstract

**Background:**

Regarding the increasing application of neuromusculoskeletal sonography among medical specialties, specifically physiatrists, this study aims to assess the knowledge and skill level of these specialists in neuromusculoskeletal sonography in Iran.

**Methods:**

This descriptive, cross-sectional study was performed in 2018. The utilized questionnaire developed based on previous studies in collaboration with 6 university lecturers of Shaheed Beheshti, Iran, and Tabriz medical universities and a physiatrist from Hacettepe University (Turkey); it included questions entailing demographic data, knowledge, and performance levels. Its validity and reliability were evaluated through face validation, pilot study, and the Cronbach α calculated via SPSS. Data extraction and analysis were also performed by SPSS-25.

**Results:**

Of 364 questionnaires distributed, 300 were properly filled and entered into the study, of which, 38% were filled by clinical residents, 10% university lecturers, and 52% other categories (e.g. private sector).

The average number of musculoskeletal patient visits was 140.6 ± 119 and the mean number of musculoskeletal sonographies requested was 8.2 ± 5.2 per month (the three most common indications reported as the shoulder, carpal tunnel syndrome, and tendon injuries).

95% of the participants considered the importance of sonography for physiatrists to be “very high” or “high”; with the most valuable applications “as a guide for procedures (90%), its diagnostic utility (68%), and follow up/evaluating the response to treatment (45%). 86% of physiatrists reported they had participated in musculoskeletal sonography courses, 60% during residency, and the rest through workshops.

Also, the participants mentioned safety (83%), the possibility of performing simultaneous diagnosis and intervention procedures (70%), repeatability (58%), and dynamic imagery (52%) as the major advantages of musculoskeletal ultrasound.

**Conclusion:**

a large number of doctors consider musculoskeletal sonography to be essential for physiatrists, though insufficient education on the subject and the low number of ultrasound devices are some of the obstacles in enhancing the use of this technology in PM&R setting. Presenting certified specific training courses during residency, provision of necessary rotations, using the capacities of the PM&R scientific committee, and the private sector for running workshops and professional training courses are suggested for enhancing the knowledge and skills of neuromusculoskeletal sonography.

## Background

Advancements in ultrasound technology, improved imaging resolution, reduction in the size of the devices, and increased portability have led to a remarkable increase in its utilization in various medical fields including PM&R. Physiatrists encounter a vast range of musculoskeletal disorders, therefor knowledge, and skill in neuromusculoskeletal sonography can have an impactful effect in enhancing the quality of their daily practice. On the other hand, in addition to being used as a diagnostic and treatment tool, it can provide the basis for further practical researches.

Also, sonography is a non-invasive diagnostic modality that is radiation-free and has no definite contraindications [1, 2]. Considering the vast usage of this method in the evaluation and diagnosis of soft tissue injuries, as well as lower cost compared to MRI, using ultrasound can reduce the costs in PM&R services [3].

Currently, the most common indications of using ultrasound in PM&R by the practitioners are as follows: as a guide in peripheral nerve block, local anesthesia and other pain control procedures, intraarticular and spinal injections (epidural block, facet joints, ganglions and …), soft tissue injections, and the diagnostic role in pathologies such as tendon, muscle, and nerve injuries [3–13]. Also, this method has been used in evaluating joint disorders such as knee osteoarthritis and temporomandibular joint derangement [14, 15].

Because of the extensive use of sonography as an effective and non-invasive diagnostic tool, it has been employed in various fields of medicine worldwide, and many studies have been performed regarding its application. Bruyn studied the benefits of ultrasound as a guide in therapeutic interventions and has stated the importance of training doctors and particularly rheumatologists in this regard [8]. Furthermore, Finnoff et al., while presenting a training program for PM&R residents in Mayo Clinic, considered training in this field to be necessary for residency programs [16]. In 2015, in a systematic review they performed, definite evidence was found concerning the higher accuracy of ultrasound-guided injections compared to landmark guided injection and they further emphasized the need for non-radiologist doctors to be trained in this area [17].

Stoven et al. [18] also suggested the addition of sonography as an important training subject for internal medicine residents. The studies performed by Coris [3], Luz [19], and Arger [20] also emphasize the effectiveness, accuracy, and cost-effectiveness of ultrasonography in the field of musculoskeletal disorders and deem it essential for physicians to receive sufficient training.

On the other hand, the utilization of neuromusculoskeletal sonography by PM&R physicians in Iran dates back to approximately 10 years ago.

In 2016, Raeissadat et al. reported ultrasound-guided intraarticular injections in the glenohumeral joint to be more accurate than landmark-guided injections [21]. Also, the study performed by Rayegani et al. (2019) suggested diagnostic ultrasound as an adjunct or alternative method for electrodiagnosis in detecting ulnar neuropathy at the elbow [22].

Over time, training for this skill has evolved from a few hour training courses into advanced several days workshops. Training for this skill has also been routinely integrated into many residency programs. Since there is not much information regarding the effectiveness of the courses in meeting the needs of the graduates as well as the limiting factors in the employment of this modality, this study aims to evaluate the level of knowledge, perspectives, and performance of Iranian PM&R practitioners regarding neuromusculoskeletal sonography.

## Methods

**Fig. 1:**
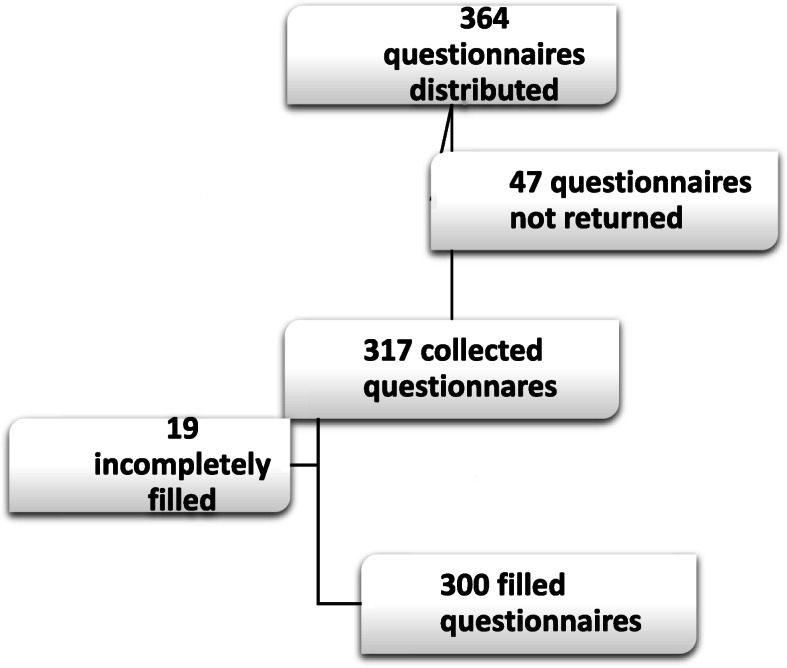
Consort chart

This descriptive cross-sectional study was performed in 2018. The questionnaire (see Additional file 1) was developed for this study and has not been published elsewhere; it includes questions concerning demographic data as well as the level of knowledge, views, and performance of the participants. This questionnaire was designed based on previous studies alongside collaboration with 6 university lecturers of Shaheed Beheshti, Iran, and Tabriz universities of medical sciences as well as a specialist in PM&R from Hacettepe University in Turkey. Its validity and reliability were first assessed using face validation by selected lecturers of these universities, and afterward through a pilot study on 40 residents and specialist physicians. Eventually, Cronbach’s alpha was calculated using SPSS 25 (> 0.7). **(**Fig. 1**).**

The questionnaires were completed by PM&R specialists and residents during the twenty-second PM&R congress and the fourth symposium on osteoporosis and arthritis in 2018; which was held by the PM&R committee of Iran. 26 questions were inquiring about the following information: demographic data, university and the year of graduation, the average number of musculoskeletal patient visits, neuromusculoskeletal sonography requests and MRI requests per month, type and duration of sonography training, level of knowledge about the fundamentals of sonography, key terms and basic concepts, the applications, advantages, and preferences of using sonography compared to other imaging methods, obstacles in the growth of using sonography among PM&R specialists, and suggestions for any improvement in this regard.

Data analysis was performed using SPSS 25. To describe qualitative data, mean, standard deviation, median, and range were used. For qualitative data, frequency and percentage of frequency were used. Tables and figures were created accordingly.

## Results

A total of 364 questionnaires were distributed, of which, 47 questionnaires were not returned, 17 were incompletely filled, and 300 were completed and entered into the study.

The mean age of the participants was 34 ± 4.45 years (between 26 to 55 years). The female to male distribution was 65.7 to 43.3%. Thirty-eight percent of the participants were residents, 10% were university lecturers, and 52% were in other sectors (private and public sector practitioners). The number of years past since the beginning of residency was 0–3 years in 38%, 4–6 years in 34%, 6–10 years in 20%, and more than 10 years in 8%.

The mean number of musculoskeletal patients visited per month by each participant was 140.6 ± 110 (minimum of 5 and a maximum of 800).

The mean of total neuromusculoskeletal sonography requests was 8.2 ± 5.2 per month. This number was 12.01 ± 1.2 for residents, 9.2 ± 1.3 for university lecturers, and 5.1 ± 2.2 for the others.

**Fig. 2:**
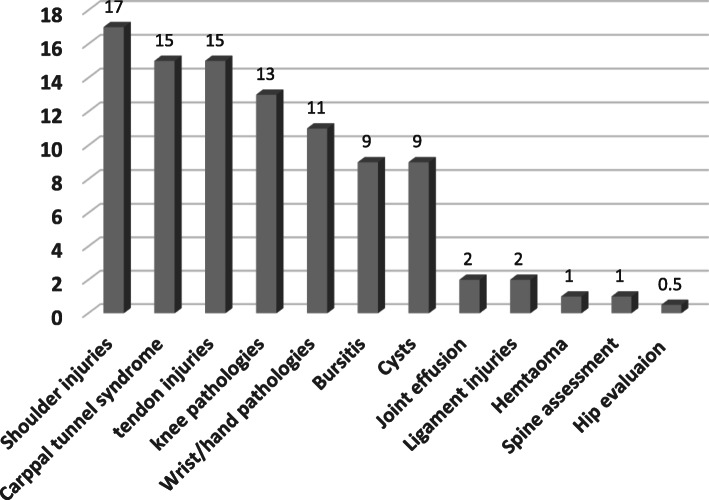
Common indication for neuromusculoskeletal sonography requests according to participants (frequency percentage)

The common indications for neuromusculoskeletal sonography requests based on the area were as follows: the shoulder joint (17%), carpal tunnel syndrome (15%), tendon injuries (15%), knee pathologies (13%), hand and wrist pathologies (11%), bursae (9%), cysts (9%), joint effusions (2%), ligament damage (2%), hematomas (1%), spine evaluation (1%), and hip evaluation (0.5%)(Fig. 2).

Forty-two percent of the participating physicians performed neural and musculoskeletal sonography during their routine practice. Among these, 66% mentioned that they used it at least once per day, and 31% of them claimed to use it at least once a week. The remaining 57% of the participants did not use this method, of which, the reasons for not performing these procedures as claimed were as follows: 65% mentioned not having an ultrasound device, 60% mentioned insufficient training, 45% not having clear charging prices for the procedures, 30% blamed lack of insurance coverage, and 5% other reasons.

It is worth mentioning that when asked about their opinion on whether neuromusculoskeletal sonography should solely be performed by radiologists, and that other physicians should not be able to perform these procedures, 70% of the participants responded “disagree” or “strongly disagree”.

**Fig. 3:**
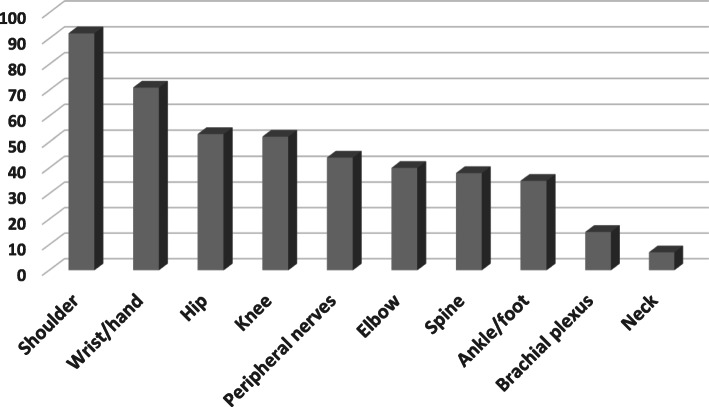
Anatomical regions in which ultrasound was considered valuable by participants (frequency percentage)

The most significant applications according to the views of the participants in descending order were as follows: guiding the therapeutic procedures (85%), diagnosis of sports injuries (48%), and diagnosis of peripheral nerve injury (37%). On the other hand, the best-visualized tissues and anatomical regions by ultrasound were reported as tendon tissue (96%), muscles (94%), and nerves (85%). As for the anatomical regions, the shoulder region (92%), wrist and hand (71%), hip (53%), knee (52%), were mentioned (Fig. 3).

**Fig. 4:**
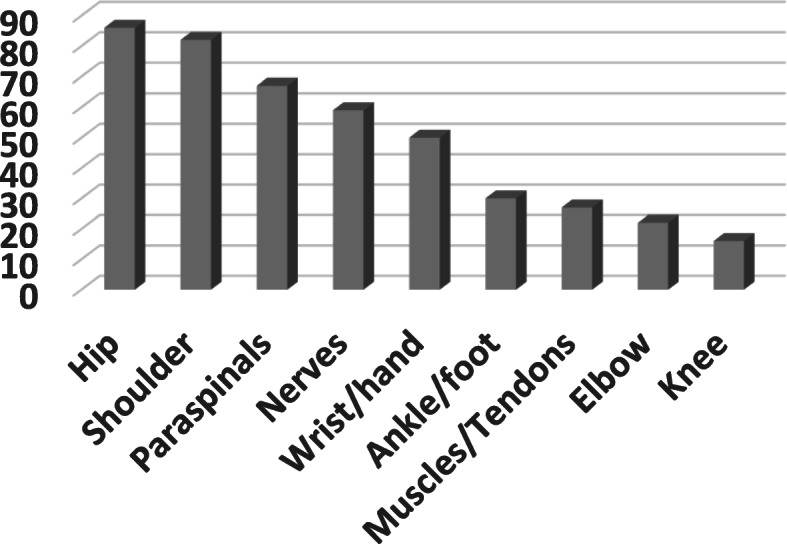
Cases in which ultrasound-assisted procedures are preferred to blind injection according to participants

The mean of total musculoskeletal MRIs requested by participants was 14 ± 8.4 cases per month. According to the participants, the anatomical regions in which ultrasound is preferred to MRI were: the shoulder (64%), peripheral nerves (55%), wrist and hand (43%). Furthermore, ultrasound-guided injections in the hip, shoulder, and spine are mentioned superior to landmark-based injections (Fig. 4).

Based on the study results, 84% of university lecturers, 67% of residents, and 79% of the other participating doctors were familiar with at least 5 of the following ultrasound specific terms: anisotropy, acoustic enhancement, reverberation through transmission, acoustic shadowing, electrographic imaging, color flow, power Doppler.

Among those who participated, 61% rated the necessity of ultrasound training for PM&R specialists as “very high” and 24% as “high”. Eighty-six percent of the physicians had participated in training courses for neuromusculoskeletal sonography, 60% of which had been during residency and the others through workshops (single or several-days-long).

Forty percent of our study population used books for learning theoretical material regarding sonography, while 26% studied the issued guideline. Ninety percent of the participants considered the application of this method in PM&R to be most valuable in guided injections and interventions, 68% for diagnostic evaluation, and 48% for follow up and assessment of response to treatment.

Besides, the advantages of using sonography compared to other imaging methods according to participants were reported as follows: safety (83%), the ability to simultaneously perform diagnostic and therapeutic interventions (70%), repeatability (58%), dynamic imaging (52%), accessibility and speed of assessment (48%), the ability to compare both sides (33%), high resolution (15%), cost efficiency (13%).

Based on the results of this study, the obstacles faced by PM&R specialists in using sonography have been reported as “no access to an ultrasound device” (65%), “lack of sufficient training” (60%), “unclear tariffs for the performed procedures” (30%), and professional issues in the workplace (5%).

## Discussion

Musculoskeletal disorders are of importance to PM&R specialists since they are the major reasons for patient visits. Therefore, applying the latest knowledge and technology in diagnosis and treatment can improve the quality of services they can provide. Considering the prominent role of sonography as a multipotent tool for PM&R practitioners, providing them with the required training is of great importance. Various studies have been performed regarding this subject in different countries, including Coris et al. [3] in 2011, which has considered sonography an effective, non-invasive modality in the assessment and diagnosis of limb injuries and have claimed it to be more cost-effective than MRI. Besides, they have stated it has considerable safety and accuracy in therapeutic interventions such as PRP, dry needling, as well as injecting steroids, hyaluronic acid, and botulinum toxin.

In a study by Bruyn et al. [8], the role of sonography as a guide in therapeutic modalities and injections (by showing the path of the needle and the possibility of marking the intended location) was assessed and the necessity of teaching this skill to physicians, and in particular rheumatologists was emphasized.

Furthermore, Finoff et al. [16] in 2010 while introducing a certified residency program for PM&R residents in Mayo Clinic, regarded this skill as essential during the residency program. This researcher also performed a systematic review in 2015, in which definite evidence of the more effectiveness, efficiency, and cost-effectiveness of ultrasound-guided injections (compared with landmark guided injections) were reported. In this study, the importance of ultrasound training for non-radiologist physicians; in particular sports medicine fellowships, was emphasized [17].

Stoven et al. [18], observed that after an ultrasound training workshop for internists, their knowledge and skill improved significantly, and using a sonography device was considered as “a skill obtainable in a short time”. They also regarded sonography training as an important field for internal medicine specialists.

The results of a study by Luz et al. [19] showed that after presenting training courses in the curriculum, the knowledge level of residents in neuromusculoskeletal sonography increased significantly, to the extent that 86% of the residents who participated in the study claimed ultrasound training to be “very necessary” or “definitely essential”. Also, 73% of them considered self-learning of this skill to be “useful” or “very useful”.

Arger et al. [18] also reported an increase in the knowledge of medical students after an ultrasound training course.

On the other hand, Delle et al. [23] conducted a study in 2008; and while emphasizing the effectiveness of sonography in the diagnosis of musculoskeletal and rheumatologic diseases (such as active inflammation in spondyloarthropathies, crystalopathies, etc.), mentioned the limitations of this method including the operator-dependency and lack of standard diagnostic protocols.

Also, D’Agostino et al. [24] in France in 2013, assessed the applications of sonography in rheumatology and reported the lack of training and specific guidelines in this field.

Nofsinger et al. [25], while pointing out the specific uses of sonography in sports medicine (including the diagnosis of rotator cuff tears, elbow ligaments damage, and intra-articular knee damage), they mentioned lack of sufficient training as one of the limiting factors in using this diagnostic modality.

In a study by Moderiano et al. [26] which evaluated the amount of knowledge, views, and performance of 47 sonography experts; reported a low level of knowledge in this study population.

Furthermore, the results of a study by Bagley et al. [27] also points out the low level of knowledge and performance quality of sonographers,

In this study, despite the difference in the study population and available facilities (the country of Iran compared to developed countries in which the abovementioned studies have been performed) the importance of employing ultrasound in the field of PM&R as a guide in interventions, follow up and assessing response to treatment has been considered. On the other hand, cost-effectiveness compared to MRI and the superiority of ultrasound-guided injections compared to landmark-based methods have been reported to be among the most important applications and benefits of sonography. This is worth to be noted that, in developing countries like Iran, not all the physicians have access to many imaging technologies (e.g. MRI as a diagnostic tool, or x-ray or fluoroscopy to navigate injections), and introducing ultrasound as an acceptable and feasible substitute method is of great importance.

As mentioned, a considerable number of participants in the current study regard neuromusculoskeletal sonography training to be essential for PM&R specialists. Despite the majority of respondents (86%) declared they have participated in some sort of ultrasound training programs, most of them mentioned insufficient training (or inadequate quality or quantity of educational courses) and inaccessibility of ultrasound devices due to financial reasons are among the most important obstacles in using this tool in the PM&R setting.

For this reason, the following suggestions have been made to improve the quality of neuromusculoskeletal sonography services:

- Presenting the neural and musculoskeletal sonography (NMSK US) course in educational curriculums as rotations.

- Empowering PM&R specialists in the field of MSK US with the PM&R committee and other educational associations’ assistance.

- Collaboration with continuous educational organizations for presenting short training courses for PM&R graduates.

- Using virtual training (video and other visual media) to train specialists in MSK US.

- Providing educational centers, books, and guidelines of MSK US.

- Coordinating with the respective organizations and medical equipment vendors about creating special provisions for purchasing sonography devices.

- The collaboration of the PM&R committee with policymakers to regulate the tariffs related to ultrasound-guided injections.

- Negotiating with insurance companies to cover ultrasound-guided injections fees.

- Using the capacities of private centers (pain clinics, PM&R clinics, and sports medicine centers) to train physicians and also to refer patients for procedures by PM&R specialists.

- Informing PM&R specialists, other physicians as well as patients about the benefits and limitations of ultrasound use in procedures.

## Conclusion

The emerging technology and widespread application of neuromusculoskeletal sonography are known to medical practitioners and specifically physiatrists. A large number of studies had shown its accuracy, safety, and cost-efficiency in different diagnostic and therapeutic procedures. Although, regarding the different education systems and facilities available in Iran, this study aimed to assess the knowledge level and educational perspectives of Iranian physiatrists. The majority of participants in this study considered MSK US as an essential and useful skill for physiatrists, although they mentioned insufficient education and inaccessible US device as the major limitations of its application in the PM&R setting.

According to these physicians, the tendon is the best-visualized tissue, and they named “shoulder” as the most agreed region in which ultrasound is preferred to MRI. Also, safety and the possibility of performing simultaneous diagnostic and therapeutic procedures were most frequently reported as the advantages of this method.

In conclusion, possible approaches to enhance the quality and accessibility of MSK ultrasound for physiatrists are mentioned, such as collaborating with PM&R committees and relevant educational associations, adjoining MSK sonography courses in the residency curriculum, and negotiating with insurance providers to facilitate and alleviate economic issues of applying of this method for both patients and physicians.

### Strengths and limitations of this study

To the best of our knowledge, this study is the first one of its kind conducted on this topic in the field of musculoskeletal medicine including physical medicine and rehabilitation.

The relatively large sample size is one of the strengths of this study. Among the 522 PM&R specialists and residents from the universities across the country, eventually, 317 (approximately 60%) participated in the study. The questionnaire used in this study was designed and evaluated by university lecturers.

The limitations of the study include some of the questions being too “obvious”, which has led to a disparity in responses. On the other hand, including questions that had the option of choosing more than one answer reduced the precision, also increasing the difficulty of final data analysis in some cases. Furthermore, to increase the study population, the PM&R specialists and residents were studied simultaneously, but the inconsistency in the responses of these two groups, in some cases led to discrepancies in the final results. To alleviate these differences, the analysis of each group was performed separately.

## Supplementary information

**Additional file 1.** The questionnaire developed and used for data collection in this study; English version.

## Data Availability

The datasets used and/or analyzed during the current study are available from the corresponding author on reasonable request.

## Abbreviations

PM&R: Physical medicine and rehabilitation
MSK: Musculoskeletal
NMSK: Neuromusculoskeletal
MRI: Magnetic resonance imaging
PRP: Platelet-rich plasma
US: Ultrasound
