# Supplementary material for: Beliefs, attitude, and knowledge of the Iranian physiatrists towards neuromusculoskeletal ultrasound and common barriers in its application
Source: BMC Musculoskelet Disord. 2020 Oct 14;21:680. doi: 10.1186/s12891-020-03708-1 (PMC7559464; doi:10.1186/s12891-020-03708-1)
Supplement: Supplementary file 1 — Additional file 1. The questionnaire developed and used for data collection in this study; English version. [file 12891_2020_3708_MOESM1_ESM.docx]

**The questionnaire**

**“Believes, attitude and knowledge of the Iranian physiatrist’s towards Neuromusculoskeletal ultrasound and common barriers in its application”**

1. Personal info:
2. Age:….
3. Gender:….
4. The university of graduation/residency:
5. Artesh university of medical sciences
6. Isfahan University of medical sciences
7. Iran University of medical sciences
8. Baqiyatallah University of medical sciences
9. Tabriz University of medical sciences
10. Shaheed Beheshti University of medical sciences
11. Shiraz University of medical sciences
12. Occupational status (more than one can be chosen)
13. Resident
14. Compulsory medical services program
15. Governmental/ public occupancy
16. Private sector occupancy
17. University faculty
18. Others: (please specify:....)
19. Years passed since the beginning of the residency:…..
20. The mean number of musculoskeletal patient visits per month (referral electrodiagnosis cases excluded):…..
21. The mean number of musculoskeletal MRI requests per month; please name the 3 most common indications:…..
22. The mean number of neuromusculoskeletal ultrasound requests per month; please name the 3 most common indications:…..
23. Do you believe that musculoskeletal ultrasound examinations should be exclusively performed by radiologists?
24. Strongly agree
25. Agree
26. Neutral
27. Disagree
28. Strongly disagree
29. Do you perform musculoskeletal ultrasound examinations in your routine daily practice?
30. No, and the reason is:
31. Not having the ultrasound device
32. Insufficient training experience
33. No clear charging prices for the procedures
34. No insurance coverage
35. Other reasons: please specify:
36. Yes:
37. At least once a day
38. At least once a week
39. At least once a month
40. Of these mentioned below, which one do you consider as an obstacle for musculoskeletal ultrasound application by physiatrists? (please number them in the order of importance)
41. No access to an ultrasound device
42. Lack of sufficient training
43. Unclear tariffs for the performed procedures
44. No insurance coverage
45. Professional considerations
46. Other reasons: please specify
47. How do you rate the necessity of musculoskeletal ultrasound education for physiatrists?
48. Very high
49. High
50. Neutral
51. Low
52. Very low
53. Have you ever been educated on the musculoskeletal ultrasound examination?
54. No
55. Yes :
56. During residency
57. Participating in a 2-day workshop
58. Participating in many 2-day workshops
59. A one-month training course
60. A 2 to 4 months training course
61. More than four months of training
62. Which one do you consider the most significant application of musculoskeletal ultrasound examination in the field of physical medicine and rehabilitation?
63. Diagnostic applications
64. Guide for injections and procedures
65. Follow up and treatment response evaluation
66. In which cases you believe applying the musculoskeletal ultrasound can offer the most valuable information? (more than one can be chosen)
67. Diagnosis of sport injuries
68. Diagnosis of Rheumatologic diseases
69. Guiding therapeutic injections/procedures
70. Diagnosis of Fractures
71. Detecting Foreign bodies
72. Diagnosis of Degenerative joint disorders
73. Detecting the Stump complications
74. Detecting Tumors
75. Diagnosis of the peripheral nerve injuries
76. Which one of these structures do you consider to be best-visualized by ultrasound? (more than one can be chosen)
77. Muscles
78. Tendons
79. Ligaments
80. Bone cortex
81. Periosteum
82. Nerves
83. Bursae
84. Cartilages/ menisci
85. Skin
86. Subcutaneous fat
87. Lymph nodes
88. Vessels
89. In which one of these anatomic regions do you consider musculoskeletal ultrasound examination to be the most informative? (more than one can be chosen)
90. Neck
91. Shoulder
92. Elbow
93. Hand/wrist
94. Hip
95. Knee
96. Foot/ankle
97. Brachial plexus
98. Peripheral nerves
99. Spine
100. In which one of below mentioned structures, do you prefer musculoskeletal ultrasound examination over MRI? (more than one can be chosen)
101. Muscles
102. Tendons
103. Ligaments
104. Bone cortex
105. Periosteum
106. Nerves
107. Bursae
108. Cartilages/menisci
109. Skin
110. Subcutaneous fat
111. Lymph nodes
112. Vessels
113. In which one of below mentioned anatomic regions, do you prefer musculoskeletal ultrasound examination over MRI? (more than one can be chosen)
114. Neck
115. Shoulder
116. Elbow
117. Hand/wrist
118. Hip
119. Knee
120. Foot/ ankle
121. Brachial plexus
122. Peripheral nerves
123. Which one do you consider as the main advantage of musculoskeletal ultrasound? (more than one can be chosen)
124. Cost efficiency
125. Safety
126. Repeatability
127. Dynamic imaging
128. Side-to-side comparability
129. High resolution
130. Accessibility and quick evaluation
131. The possibility of performing simultaneous diagnostic and therapeutic interventions
132. In which one of these anatomical regions do you prefer to perform ultrasound-guided injection over landmark-guided injection? (more than one can be chosen)
133. Shoulder
134. Elbow
135. Wrist
136. Hand
137. Hip
138. Knee
139. Ankle
140. Foot
141. Paraspinals
142. Muscles and tendons
143. Nerve proximities
144. Have you ever studied a textbook on musculoskeletal ultrasound?
145. No
146. Yes; (Please name the one)
147. Have you ever studied guidelines on musculoskeletal ultrasound?
148. No
149. Yes; (Please name the one)
150. Which one of the following phrases are you familiar with?
151. Anisotropy
152. Acoustic enhancement
153. Reverberation Through transmission
154. Acoustic shadowing
155. Elastographic imaging
156. Color flow
157. Power Doppler
158. Which means of musculoskeletal ultrasound education do you prefer/suggest?
159. Daily sessions
160. Weekly sessions
161. Monthly sessions
162. Few-day workshops
163. Rotation course during residency
164. Which one of these following settings/tools of the ultrasound equipment are you familiar with? (more than one can be chosen)
165. Transducer selection
166. Depth
167. Focal zone
168. Gain
169. Time gain compensation
170. Zoom
